# ﻿*Melomastia* (Dothideomycetes, Ascomycota) species associated with Chinese *Aquilaria* spp.

**DOI:** 10.3897/mycokeys.111.137898

**Published:** 2024-12-04

**Authors:** Tian-Ye Du, Samantha C. Karunarathna, Saowaluck Tibpromma, Kevin D. Hyde, Somrudee Nilthong, Ausana Mapook, Xiang-Fu Liu, Dong-Qin Dai, Chen Niu, Abdallah M. Elgorban, Ekachai Chukeatirote, Hao-Han Wang

**Affiliations:** 1 Research Center of Natural History and Culture, Center for Yunnan Plateau Biological Resources Protection and Utilization, Key Laboratory of Yunnan Provincial Department of Education of the Deep-Time Evolution on Biodiversity from the Origin of the Pearl River, College of Biological Resource and Food Engineering, Qujing Normal University, Qujing 655011, China; 2 Center of Excellence in Fungal Research, Mae Fah Luang University, Chiang Rai 57100, Thailand; 3 School of Science, Mae Fah Luang University, Chiang Rai 57100, Thailand; 4 Spice and Beverage Research Institute, Chinese Academy of Tropical Agriculture Sciences, Wanning 571533, China; 5 Department of Botany and Microbiology, College of Science, King Saud University, Riyadh 11451, Saudi Arabia

**Keywords:** 2 new species, Dyfrolomycetales, new records, Pleurotremataceae, saprobes, Thymelaeaceae

## Abstract

This study is based on three terrestrial saprobic fungi associated with *Aquilaria* in Guangdong and Yunnan provinces in China. All isolated species matched with generic concepts of *Melomastia*. Detailed morphological characteristics and combined multigene phylogeny of LSU, SSU, and TEF revealed that the new isolates represent two new species (*Melomastiaguangdongensis* and *M.yunnanensis*), and one new host and geographical record (*M.sinensis*). *Melomastiaguangdongensis* is distinct from the phylogenetically closest species in having semi-immersed to immersed, globose to subglobose ascomata, and two strata of the peridium. *Melomastiayunnanensis* differs from the phylogenetically closest species in having immersed ascomata, conical ostiolar canals, and branched pseudoparaphyses. The discovery of these two new species and one new record collected expands the number of saprobic species associated with *Aquilaria* from 28 to 31. Descriptions, photo plates, and phylogenetic analyses of taxa are provided.

## ﻿Introduction

Pleurotremataceae Walt. Watson was introduced by Watson (1929) to accommodate *Pleurotrema* Müll. Arg. with *P.polysemum* (Nyl.) Müll. Arg. as the type species. The familial placement of *Pleurotrema* has been controversial, as the mature asci are neither typically unitunicate nor bitunicate (Mathiassen 1989; Hyde 1992). The placement of *Pleurotrema* has been confirmed based on the re-examined feature of the type of species *P.polysemum*, and Maharachchikumbura et al. (2016) transferred Pleurotremataceae from Sordariomycetes O.E. Erikss. & Winka to Dothideomycetes O.E. Erikss. & Winka and synonymized Dyfrolomycetaceae K.D. Hyde, K.L. Pang, Alias, Suetrong & E.B.G. Jones under Pleurotremataceae based on morphological comparison. Currently, Pleurotremataceae is accepted as the type and only family in Dyfrolomycetales K.L. Pang, K.D. Hyde & E.B.G. Jones, with three genera, *Dyfrolomyces* K. D. Hyde, *Melomastia* Nitschke ex Sacc, and *Pleurotrema* in this family (Maharachchikumbura et al. 2016; Hongsanan et al. 2020; Wijayawardene et al. 2022; Hyde et al. 2024).

*Melomastia* was established by Saccardo (1875) to accommodate *M.mastoidea* (Fr.) J. Schröt. (=*Melomastiafriesii* Nitschke) as the type species. Previously, relying solely on the morphological features of *Melomastia* type species, the genus was considered unresolved and classified under Ascomycota genera *incertae sedis* (Maharachchikumbura et al. 2016). Subsequently, Norphanphoun et al. (2017) assigned *Melomastia* to Pleurotremataceae based on the newly introduced taxon *M.italica* Norph., Camporesi, T.C. Wen & K.D. Hyde, supported by sequence data. Based on morphology and phylogenetic analyses, Li et al. (2022) synonymized *Dyfrolomyces* under *Melomastia* and simultaneously transferred 11 species from *Dyfrolomyces* to *Melomastia*. De Silva et al. (2022) reported two new records of *Melomastia* from Thailand. However, Kularathnage et al. (2023) maintained *Dyfrolomyces* to accommodate *D.tiomanensis* K.L. Pang, Alias, K.D. Hyde, Suetrong & E.B.G. Jones and *D.chromolaenae* Mapook & K.D. Hyde, based on morphology differences of ascospores and the phylogenetic analyses. Recently, some new taxa from Brazil, China and Thailand have been introduced, *viz. M.beihaiensis* T.Y. Du, K.D. Hyde & Tibpromma (Senanayake et al. 2023), *M.loropetalicola* Kular., W. Dong & K.D. Hyde (Dong et al. 2023), *M.puerensis* R.F. Xu & Tibpromma (Xu et al. 2024), *M.pyriformis* Kular. & Senan. (Kularathnage et al. 2023), *M.septata* J.Y. Zhang, K.D. Hyde & Y.Z. Lu (Hyde et al. 2023), and *M.septemseptata* Muxfeldt & Aptroot (Muxfeldt Naziazeno and Aptroot 2023). Currently, 66 epithets of *Melomastia* are listed in Index Fungorum (2024), while only 20 species have sequences available in GenBank.

*Melomastia* is characterized by immersed to semi-immersed, globose to subglobose, coriaceous to carbonaceous, ostiolate ascomata, dark brown peridium, filamentous pseudoparaphyses, bitunicate, cylindrical, 8-spored asci, and ascospores are fusiform to oblong, ovoid, or cylindrical, hyaline, 1–10-septate, with rounded or acute ends, with or without gelatinous sheath; while, the asexual morph of *Melomastia* is undetermined (Dayarathne et al. 2020; de Silva et al. 2022; Li et al. 2022; Kularathnage et al. 2023; Xu et al. 2024). Most *Melomastia* species have been recorded as saprobes from various habitats, such as terrestrial, freshwater, marine, and mangrove ecosystems (Hyde 1992; Hyde et al. 2017; Norphanphoun et al. 2017; Dayarathne et al. 2020; Phukhamsakda et al. 2020; Li et al. 2022; Hyde et al. 2023; Tian et al. 2024; Xu et al. 2024). *Melomastia* is a geographically widely distributed genus with a broad host range, which has been systematically documented in Li et al. (2022) and Kularathnage et al. (2023), *viz.* members of *Melomastia* have wide geographical distribution in Africa, Asia, Australia, Europe, and South America, while the reported hosts of *Melomastia* belong to Acanthaceae Juss., Asteraceae Bercht. & J. Presl, Euphorbiaceae Juss., Hamamelidaceae R. Br., Oleaceae Hoffmanns. & Link, Ranunculaceae Juss., Rhizophoraceae Pers., Theaceae Mirb., and Vitaceae Juss.

*Aquilaria* Lam. is an important agarwood resin-producing tree genus in Thymelaeaceae Juss. Agarwood resin is high-valued and very rare, and its formation is primarily due to injury, followed by microbial infection (Rasool and Mohamed 2016; Azren et al. 2018; Wang et al. 2018). So far, many reports have been published on the pathogenic and endophytic fungi associated with *Aquilaria*, while saprobic fungi have been neglected (Liu et al. 2020; Du et al. 2022a). Prior to 2022, there were only eight records of saprobic fungi associated with *Aquilaria* (Punithalingam and Gibson 1978; Subansenee et al. 1985), and molecular data and comprehensive morphological descriptions were lacking. Recently, 20 saprobic fungal species have been reported from *Aquilaria* spp. by Du et al. (2022b, 2023, 2024), Chethana et al. (2023), and Hyde et al. (2023, 2024) based on both morphological and molecular evidence. Therefore, so far only 28 records of *Aquilaria*-associated fungi have been found. This study focuses on filling the gap in research on the saprobic fungi associated with *Aquilaria*, and enriching the diversity of fungi associated with *Aquilaria*.

In this study, *Aquilaria* plant specimens with black ascomycetous fungal fruiting bodies were collected from Yunnan and Guangdong provinces in China. Based on phylogenetic and morphological analyses, these fungal collections were identified as two new species and one new record of the *Melomastia*. Full descriptions, illustrations, photo plates, and phylogenetic trees to indicate the placement of new taxa are provided.

## ﻿Materials and methods

### ﻿Sampling, examination, and isolation

Dead fallen branches of *Aquilaria* spp. with ascomycetous fungal fruiting bodies were collected from subtropical parts of Guangdong and Yunnan provinces in China. After recording important information (Rathnayaka et al. 2024), samples were transported to the laboratory in plastic bags. Morphological structures were examined by using an OPTEC SZ650 dissecting stereomicroscope (Chongqing, China), and an OLYMPUS DP74 (Tokyo, Japan) digital camera on an OLYMPUS optical microscope (Tokyo, Japan) was used to observe and photograph the microstructure of fungi. Micro-morphological structures were measured in Tarosoft ® Image Framework program v. 1.3, and photo plates were edited in Adobe Photoshop CS3 Extended version 22.0.0 software (Adobe Systems, California, the USA).

Fungi were isolated using single-spore isolation, as described by Senanayake et al. (2020). The fruiting bodies were cut by sterilized blades, and the ascospores were picked up by sterilized needles and cultured in potato dextrose agar (PDA) at 23–28 °C for 24–48 hours. The single germinated ascospores were picked up and transferred to PDA at 23–28 °C with recording culture characters.

Specimens were deposited at the Guizhou Medical University (GMB-W) and Mycological Herbarium of Zhongkai University of Agriculture and Engineering (MHZU), China. Living cultures are deposited in the Guizhou Medical University Culture Collection (GMBCC), Guizhou Culture Collection (GZCC), and Zhongkai University of Agriculture and Engineering Culture Collection (ZHKUCC), China. Facesoffungi (FoF) numbers were registered as described in Jayasiri et al. (2015), and MycoBank numbers (MB) were registered as outlined in MycoBank (2024).

### ﻿DNA extraction, PCR amplification, and sequencing

Molecular studies were carried out according to Dissanayake et al. (2020). Total genomic DNA was extracted from one-month-old fresh fungal mycelium (grew on PDA) using a DNA Extraction Kit-BSC14S1 (BioFlux, Hangzhou, P.R. China) following the manufacturer’s instructions. Polymerase chain reactions (PCR) were carried out using the following primers: 28S nrRNA gene (LSU) was amplified by using the primers LR0R and LR5 (Vilgalys and Hester 1990), 18S ribosomal RNA (SSU) was amplified using the primers NS1 and NS4 (White et al. 1990), and translation elongation factor 1-alpha (TEF) was amplified using the primers EF1-983F and EF1-2218R (Rehner 2001). The DNA amplification procedure was performed by PCR in a 25 μL containing 12.5 μL 2×Master Mix (mixture of Easy Taq TM DNA Polymerase, dNTPs, and optimized buffer (Beijing Trans Gen Biotech Co., Chaoyang District, Beijing, China)), 8.5 μL ddH_2_O, 2 μL of DNA template, and 1 μL of each forward and reverse primer (10 pM). The PCR thermal cycle programs for LSU, SSU, and TEF were as follows: an initialization step of 94 °C for 3 min, followed by 35 cycles of 94 °C for 30 s, an annealing step at 55 °C for 50 s, an elongation step at 72 °C for 1 min and a final extension step of 72 °C for 10 min. Purification and sequencing of PCR products were carried out by Sangon Biotech Co., Kunming, China.

### ﻿Phylogenetic analyses

A combined gene dataset of LSU, SSU, and TEF was used for the phylogenetic analyses. Newly generated contigs were used to carry out the BLASTn search in NCBI to identify the most similarities taxa of our strains. The additional sequences included in the analysis were collected from previous publications (Li et al. 2022; Kularathnage et al. 2023; Xu et al. 2024) and downloaded from GenBank (Benson et al. 2014). Phylogenetic analyses were carried out with 50 sequences (Table 1). The FASTA file used for constructing the Randomized Accelerated Maximum Likelihood (RAxML) and Bayesian Inference analyses (BI) was performed using the OFPT (Zeng et al. 2023) with the protocol. Then, the FASTA file was converted to PHYLIP and NEXUS formats for RAxML and BI phylogenetic analyses in ALTER, respectively (Glez-Peña et al. 2010).

**Table 1.** Taxa names, strain numbers, and corresponding GenBank accession numbers of the taxa included in the present study.

Taxa Names	Strain Numbers	GenBank Accession Numbers		
		LSU	SSU	TEF
*Acrospermumadeanum*	M133	EU940104	EU940031	—
*Anisomeridiumphaeospermum*	MPN539	JN887394	JN887374	JN887418
*A.ubianum*	MPN94	—	JN887379	JN887421
*Dyfrolomyceschromolaenae*	MFLUCC 17-1434 ^T^	KY111905	MT214413	MT235800
*D.tiomanensis*	MFLUCC 13-0440 ^T^	KC692156	KC692155	KC692157
*Melomastiaaquilariae*	ZHKUCC 23-0073 ^T^	OR807856	OR807854	OR832867
*M.aquilariae*	ZHKUCC 23-0088	OR807857	OR807855	OR832868
*M.beihaiensis*	KUMCC 21-0084 ^T^	MZ726990	MZ727002	OK043822
*M.clematidis*	MFLUCC 17-2092 ^T^	MT214607	MT226718	MT394663
*M.distoseptata*	MFLUCC 21-0102	MT860427	—	—
*M.fulvicomae*	MFLUCC 17-2083 ^T^	MT214608	MT226719	MT394664
*M.fusispora*	CGMCC 3.20618 ^T^	OK623464	OK623494	OL335189
*M.fusispora*	UESTCC 21.0001	OK623465	OK623495	OL335190
***M.guangdongensis***	**GMBCC1046 ^T^**	**PQ530970**	**PQ530975**	**PQ559185**
***M.guangdongensis***	**ZHKUCC 23-0040**	**PQ530971**	**PQ530976**	**PQ559186**
*M.italica*	MFLUCC 15-0160 ^T^	MG029458	MG029459	—
*M.loropetalicola*	ZHKUCC 22-0174 ^T^	OP791870	OP739334	—
*M.maolanensis*	GZCC 16-0102 ^T^	—	—	KY814762
*M.maomingensis*	ZHKUCC 23-0038 ^T^	PP809724	PP809704	PP812255
*M.maomingensis*	GZCC 23-0619	PP809725	PP809705	PP812256
*M.neothailandica*	MFLU 17-2589 ^T^	MN017857	—	—
*M.oleae*	CGMCC 3.20619 ^T^	OK623466	OK623496	OL335191
*M.oleae*	UESTCC 21.0003	OK623467	OK623497	OL335192
*M.oleae*	UESTCC 21.0005	OK623468	OK623498	OL335193
*M.oleae*	UESTCC 21.0006	—	OK623499	OL335194
*M.phetchaburiensis*	MFLUCC 15-0951 ^T^	MF615402	MF615403	—
*M.puerensis*	ZHKUCC 23-0802 ^T^	OR922309	OR922340	OR966284
*M.puerensis*	ZHKUCC 23-0803	OR922310	OR922341	OR966285
*M.pyriformis*	ZHKUCC 22-0175 ^T^	OP791870	OP739334	OQ718392
*M.rhizophorae*	BCC15481	—	KF160009	—
*M.rhizophorae*	JK 5456A	GU479799	—	GU479860
*M.septata*	MFLUCC 22-0112 ^T^	OP749870	—	OP760198
*M.sichuanensis*	CGMCC 3.20620 ^T^	OK623469	OK623500	OL335195
*M.sichuanensis*	UESTCC 21.0008	OK623470	OK623501	OL335196
*M.sinensis*	MFLUCC 17-1344 ^T^	MG836699	MG836700	—
*M.sinensis*	MFLUCC 17-2606	OL782048	—	OL875098
*M.sinensis*	MFLU 17-0777	NG_064507	—	—
***M.sinensis***	**GMBCC1008**	**PQ530972**	**PQ530977**	**PQ559187**
*M.thailandica*	MFLU 17-2610	MN017858	MN017923	MN077069
*M.thamplaensis*	KUMCC 21-0671	OQ170875	OQ168226	OR613415
*M.thamplaensis*	MFLUCC 15-0635 ^T^	KX925435	KX925436	KY814763
*M.winteri*	CGMCC 3.20621	OK623471	OK623502	OL335197
***M.yunnanensis***	**GMBCC1009 ^T^**	**PQ530973**	**PQ530978**	**PQ559188**
***M.yunnanensis***	**GZCC 23-0621**	**PQ530974**	**PQ530979**	**PQ559189**
*Muyocopronheveae*	MFLUCC 17-0066 ^T^	MH986832	MH986828	—
*Mu.lithocarpi*	MFLUCC 14-1106 ^T^	KU726967	KU726970	MT136755
*Palawaniathailandense*	MFLU 16-1873	KY086494	—	—
*P.thailandense*	MFLUCC 14-1121 ^T^	KY086493	KY086495	—
*Stigmatodiscusoculatus*	AP161116	—	—	MH756086
*S.oculatus*	AP171116	—	—	MH756087

Remarks: The newly generated sequences are indicated in bold, the superscript ^T^ indicates ex-type, and “—” indicates information unavailable.

CIPRES Science Gateway platform was used to carry out the Randomized Accelerated Maximum Likelihood (RAxML) and Bayesian Inference analyses (BI) (Miller et al. 2010). The RAxML tree analyzed with 1,000 bootstrap replicates was generated using RAxML-HPC2 on XSEDE (8.2.12) (Stamatakis et al. 2008; Stamatakis 2014) with GTR+I+G model of evolution and bootstrap supports. The BI tree was performed with MrBayes on XSEDE (3.2.7a) (Ronquist et al. 2012) by the Markov Chain Monte Carlo (MCMC) method to evaluate posterior probabilities (BYPP) (Richard and Lippmann 1991; Rannala and Yang 1996; Zhaxybayeva and Gogarten 2002). The best-fit nucleotide substitution models for each dataset were then selected based on the Bayesian information criterion (BIC) from twenty-two common DNA substitution models with rate heterogeneity by ModelFinder (Kalyaanamoorthy et al. 2017). The best model for LSU was TN+F+G4, TIM2e+I for SSU, and TN+F+I+G4 for TEF. Six simultaneous Markov chains were run for 2,000,000 generations, and a tree was sampled every 100^th^ generation. The phylogenetic tree was visualized in FigTree v.1.4.2 (Rambaut 2012), and edited by Microsoft Office PowerPoint 2021 and Adobe Photoshop CS3 Extended version 22.0.0 software (Adobe Systems, California, the USA). All newly generated sequences in this study were deposited to the GenBank (https://www.ncbi.nlm.nih.gov/WebSub/?form=history&tool=genbank).

## ﻿Results

### ﻿Phylogenetic analyses

**Figure 1.**
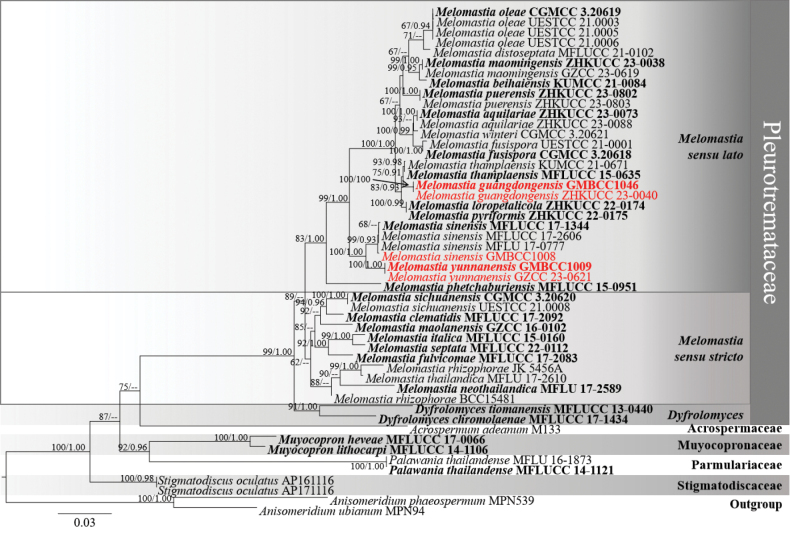
Phylogram generated from maximum likelihood analysis based on combined LSU, SSU, and TEF sequence data of 50 taxa. Bootstrap support values for maximum likelihood (ML) equal to or greater than 60% and clade credibility values greater than 0.90 from Bayesian inference analysis are labelled at each node. The tree is rooted with *Anisomeridiumphaeospermum* (MPN539) and *A.ubianum* (MPN94). The new isolates are indicated in red, and the ex-type strains are in bold.

The phylogenetic trees obtained from RAxML and BI analyses provided essentially similar topologies. The RAxML analyses of the combined dataset yielded the best scoring tree (Fig. 1), which comprised 2912 base pairs of LSU = 899, SSU = 1069, and TEF = 944. The final ML optimization likelihood value was -11933.909808. The matrix had 871 distinct alignment patterns, with 23.14% being undetermined characters or gaps. Parameters for the GTR+I+G model of the combined LSU, SSU, and TEF were as follows: estimated base frequencies A = 0.239382, C = 0.262893, G = 0.291472, T = 0.206253; substitution rates AC = 0.831502, AG = 1.991603, AT = 1.062650, CG = 0.930785, CT = 8.413262, GT = 1.000000; proportion of invariable sites I = 0.495458; and gamma distribution shape parameter *α* = 0.612808. The final RAxML tree is shown in Fig. 1.

In this phylogenetic tree, *Melomastia* was resolved as two clades, *Melomastiasensu lato* (15 species) and *Melomastiasensu stricto* (nine species), and the results are similar to those reported by Li et al. (2022), Kularathnage et al. (2023), and Xu et al. (2024). Kularathnage et al. (2023) have separated *Melomastia* into two clades, *Melomastiasensu lato*, and *Melomastiasensu stricto*; this was done due to *Melomastiasensu stricto*’s close resemblance to the type species *M.mastoidea*, while *Melomastiasensu lato* still needs more collections, sequences, and phenotypic data to support Kularathnage et al. (2023). Our two new species, *M.guangdongensis* (GMBCC1046 and ZHKUCC 23-0040) and *M.yunnanensis* (GMBCC1009 and GZCC 23-0621), and a new record *M.sinensis* (GMBCC1008) clustered within *Melomastiasensu lato.*

New species *M.guangdongensis* (GMBCC1046 and ZHKUCC 23-0040) was well separated from *M.thamplaensis* (KUMCC 21-0671 and MFLUCC 15-0635) in an independent lineage with 75% ML/0.91 PP statistical support; *M.yunnanensis* (GMBCC1009 and GZCC 23-0621) was well separated from *M.sinensis* (GMBCC1008, MFLU 17-0777, MFLUCC 17-1344 and MFLUCC 17-2606) in a distinct lineage with 100% ML/1.00 PP statistical support. The new record *M.sinensis* (GMBCC1008) was grouped within three strains of *M.sinensis* with 99% ML/0.93 PP statistical support.

### ﻿Taxonomy

#### ﻿ Melomastia guangdongensis

Taxon classificationFungiDyfrolomycetalesPleurotremataceae

T.Y. Du, K.D. Hyde, Tibpromma & Karun. sp. nov.

FC43C186-A646-5A67-8D09-4B5E3888DBFE

856407

Facesoffungi Number: FoF16958

Fig. 2

##### Etymology.

Named after the type locality “Guangdong, China”.

##### Holotype.

MHZU 23-0021

##### Description.

***Saprobic*** on a dead branch of *Aquilariasinensis*. ***Sexual morph*: *Ascomata*** (excluding neck) 180–360 µm high × 200–300 µm diam. (x– = 267 × 245 µm, n = 10), visible as black dots on the host surface, black, solitary, scattered to gregarious, semi-immersed to immersed, uniloculate, globose to subglobose, coriaceous to carbonaceous, ostiolate. ***Ostiolar canal*** 190–240 µm high × 120–160 µm wide (x– = 214 × 140 µm, n = 10), central, black, cylindrical, coriaceous to carbonaceous, filled with hyaline cells. ***Peridium*** 30–60 µm wide (x– = 40 µm, n = 20), comprising dense, several layers, outer layers brown to dark brown, thick-walled cells of ***textura angularis*** to ***textura globulosa***, inner layers hyaline, thin-walled cells of ***textura angularis*** to ***textura prismatica***, not fusion well with host tissue. ***Hamathecium*** comprising 1.5–3 µm wide, numerous filamentous, filiform, septate, sometimes branched, hyaline, pseudoparaphyses, attached to the base and between the asci, embedded in a gelatinous matrix. ***Asci*** 120–168 × 5.5–7.5 µm (x– = 144 × 6.5 µm, n = 30), bitunicate, 8-spored, cylindrical, short pedicel, rounded in apex, with an obvious ocular chamber. ***Ascospores*** (18.7–)20–26 × 5–7 µm (x– = 23 × 6 µm, n = 30), overlapping-uniseriate, hyaline, 3-septate at maturity, fusiform with acute ends, slightly constricted at the middle septum, smooth-walled, not surrounded by a mucilaginous sheath. ***Asexual morph***: Undetermined.

##### Culture characteristics.

Ascospores germinated on PDA after 24 hours, germ tubes were produced from both ends. ***Colonies*** on PDA reaching 3 cm diam., after two weeks at 23–28 °C. Colonies obverse: dense, circular, white, velvety, slightly raised at the center, entire edge. Colonies reverse: yellow, cream at the margin.

##### Material examined.

China • Guangdong Province, Maoming City, Dianbai District, Poxin, 21°34'28"N, 111°7'39"E, on a dead branch of *Aquilariasinensis* (Thymelaeaceae), 3 June 2022, T.Y. Du, MMA14, (MHZU 23-0021, holotype), ex-type, GMBCC1046, other living culture, ZHKUCC 23-0040.

**Figure 2.**
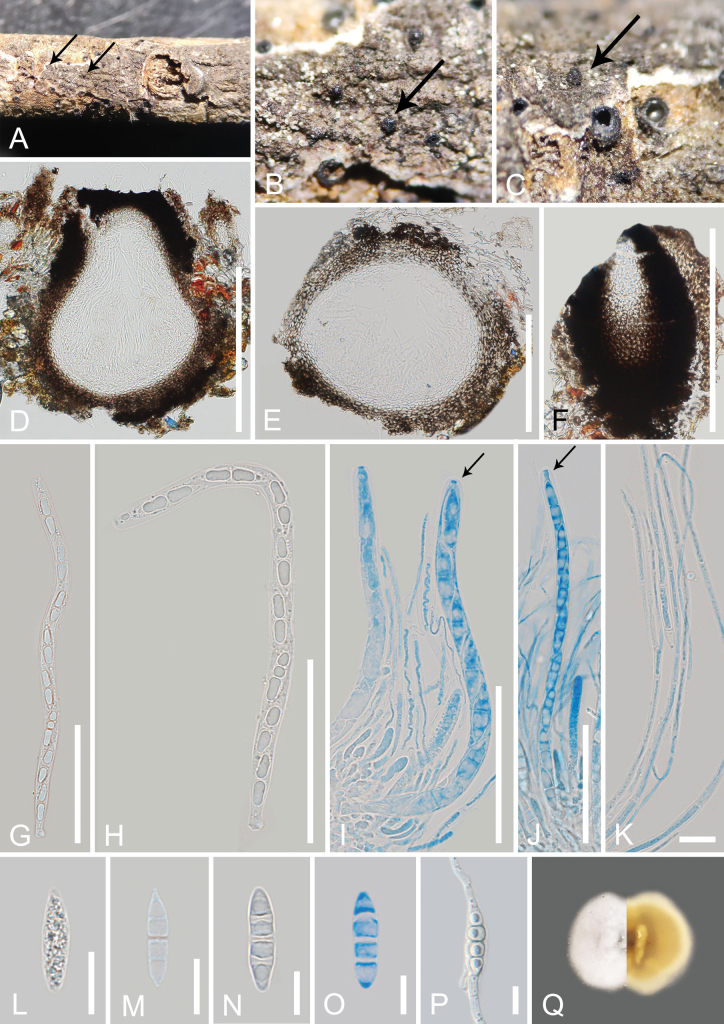
*Melomastiaguangdongensis* (MHZU 23-0021, holotype) **A–C** appearance of ascomata on the host (the arrows indicate ascomata) **D, E** vertical sections through the ascomata **F** ostiole **G–J** asci (**I, J** asci stained with cotton blue, and arrows indicate ocular chambers) **K** pseudoparaphyses stained with cotton blue **L–O** ascospores (O ascospore stained with cotton blue) **P** germinated ascospore **Q** colony on PDA obverse and reverse view. Scale bars: 200 µm (**D–F**); 50 µm (**G–J**); 10 µm (**K–P**).

##### Notes.

In the phylogenetic analyses, our new collection, *M.guangdongensis* formed a sister branch with *M.thamplaensis* strains (HKAS122773, KUMCC 21-0671, and MFLUCC 15-0635) in *Melomastiasensu lato* clade (Fig. 1) with a 75% ML/0.91 PP bootstrap support. NCBI BLASTn searches of our collection, *M.guangdongensis* showed 99.88% similarity to *M.thamplaensis* (HKAS122773) in the LSU sequence, 100% similarity to *M.thamplaensis* (AND9) in the SSU sequence, and 98.17% similarity to *M.thamplaensis* (KUMCC 21-0671) in the TEF sequence. Our new collection, *M.guangdongensis* shares similar morphology with *M.thamplaensis* in the shape of asci and ascospores. However, *M.thamplaensis* differs from *M.guangdongensis* in having clypeate, raised spots, immersed, subglobose to obpyriform, some with broad, flattened base ascomata, and three strata of peridium (Zhang et al. 2017), while *M.guangdongensis* has semi-immersed to immersed, globose to subglobose ascomata, and two strata of peridium. Base pair differences of the LSU and SSU genes between our new collection *M.guangdongensis* (GMBCC1046, ex-type) and *M.thamplaensis* (MFLUCC 15-0635, ex-type) showed that there are no nucleotide differences, while the TEF has 1.6% nucleotide differences (14/865 bp, without gaps), and a comparison of the TEF nucleotides between new collections and another strain of *M.thamplaensis* (KUMCC 21-0671) resulted in 1.7% differences (15/865 bp, without gaps) (Zhang et al. 2017; Ren et al. 2024). Therefore, we introduce our collection, *M.guangdongensis*, as a new species on a dead branch of *Aquilariasinensis* from terrestrial habitats in China, based on both morphology and phylogenetic analyses following the guidelines of Maharachchikumbura et al. (2021).

#### ﻿ Melomastia sinensis

Taxon classificationFungiDyfrolomycetalesPleurotremataceae

(Samarak., Tennakoon & K.D. Hyde) W.L. Li, Maharachch. & Jian K. Liu (2022)

9B0FC061-6CC9-5CBF-ADF5-FE602CF3EEF3

842093

Facesoffungi Number: FoF03935

Fig. 3

##### Description.

***Saprobic*** on a dead branch of *Aquilaria* sp. ***Sexual morph*: *Ascomata*** (excluding neck) 400–600 µm high × 430–580 µm diam. (x– = 515 × 520 µm, n = 10), solitary, scattered to gregarious, semi-immersed to immersed, erumpent through host tissue, globose to subglobose, black, coriaceous to carbonaceous, ostiolate. ***Ostiolar canal*** 230–365 µm high × 200–260 µm wide (x– = 303 × 230 µm, n = 10), central, black, conical, coriaceous to carbonaceous, filled with hyaline sparse periphyses. ***Peridium*** 30–120 µm wide (x– = 75 µm, n = 20), comprising dense, several layers of thick-walled cells of ***textura angularis*** to ***textura prismatica***, outer layers brown to dark brown, becoming lighter inwardly. ***Hamathecium*** comprising 2.5–6.5 µm wide, numerous filamentous, filiform, septate, unbranched, hyaline pseudoparaphyses, attached to the base and between the asci, embedded in a gelatinous matrix. ***Asci*** 175–220 × 8.5–11.5 µm (x– = 195 × 10.5 µm, n = 30), bitunicate, 8-spored, cylindrical, long pedicel, thickened and rounded apex, with an obvious ocular chamber. ***Ascospores*** (17.5–)20–26.5 × 7–9 µm (x– = 24 × 8 µm, n = 30), overlapping-uniseriate, hyaline, when ascospores gather together, they appear light yellow, mostly 6–7-septate at maturity, cylindrical, with rounded ends, slightly constricted at the septum, often similar width of cells with several small guttules, not surrounded by a mucilaginous sheath. ***Asexual morph***: Undetermined.

##### Culture characteristics.

Ascospores germinated on PDA after 24 hours, germ tubes were produced from most cells, germinated ascospores appear light yellow. ***Colonies*** on PDA reaching 3 cm diam., after two weeks at 23–28 °C. Colonies obverse: dense, circular or irregular, umbonate, cream, light yellow at the center, entire or undulate edge. Colonies reverse: dark gray, yellow at the margin.

**Figure 3.**
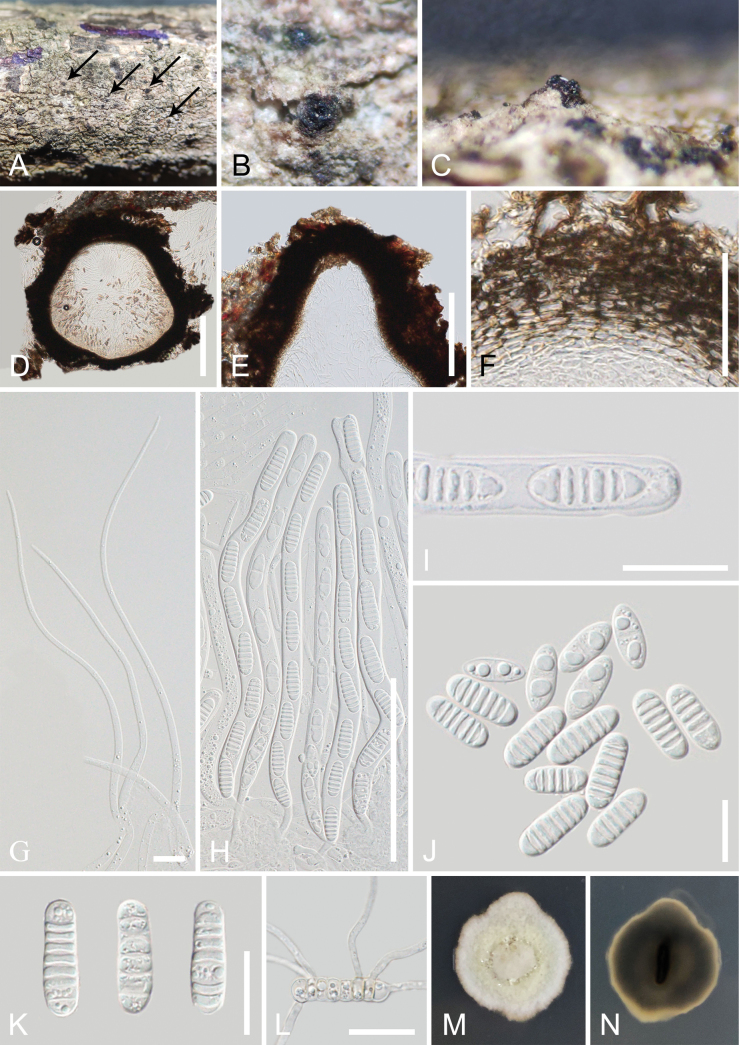
*Melomastiasinensis* (GMB-W 1006, new host and geographical record) **A–C** appearance of ascomata on the host (A the arrows indicate ascomata) **D** vertical sections through the ascoma **E** ostiole **F** peridium **G** pseudoparaphyses **H** asci **I** ascus with an ocular chamber **J, K** ascospores **L** germinated ascospore **M, N** colony on PDA obverse and reverse view. Scale bars: 200 µm (**D, E**); 100 µm (**H**); 50 µm (**F**); 20 µm (**I–L**); 10 µm (**G**).

##### Material examined.

China • Yunnan Province, Xishuangbanna, Jinghong City, Naban River Nature Reserve, 22°7'48"N, 100°40'24"E, on a dead branch of *Aquilaria* sp. (Thymelaeaceae), 14 September 2021, Tianye Du, YNA41 (GMB-W 1006, new host and geographical record), living culture, GMBCC1008.

##### Host and distribution.

*Aquilaria* sp. (China; this study), *Camelliasinensis* (Thailand; Hyde et al. 2018), and *Heveabrasiliensis* (Thailand; Senwanna et al. 2021).

##### Notes.

In the phylogenetic analyses, our new collection (GMBCC1008) isolated from a dead branch of *Aquilaria* sp. grouped with *Melomastiasinensis* strains (MFLUCC 17-1344, MFLUCC 17-2606 and MFLU 17-0777) in *Melomastiasensu lato*, with a 99% ML/0.93 PP bootstrap support (Fig. 1). NCBI BLASTn searches of our collection showed 99.78% similarity to *M.sinensis* (MFLUCC 17-2606) in the LSU sequence, 99.21% similarity to *M.oleae* (UESTCC 21.0006) in the SSU sequence, and 99.67% similarity to *M.sinensis* (MFLUCC 17-2606) in the TEF sequence.

*Melomastiasinensis* (=*Dyfrolomycessinensis* Samarak., Tennakoon & K.D. Hyde) was introduced by Hyde et al. (2018) as a saprobic on *Camelliasinensis* (L.) Kuntze stems. Our new collection shares a similar morphology with *M.sinensis* (MFLU 17-0777, holotype) in cylindrical ascospores with 6–7-septate ascospores. Our new collection has semi-immersed to immersed ascomata, differs from *M.sinensis* (MFLU 17-0777, holotype) in having superficial ascomata (Hyde et al. 2018) and differs from immersed ascomata in *M.sinensis* (MFLU 19-0232) (Senwanna et al. 2021). However, the nucleotide base pair differences between our new collection (GMBCC1008) and *M.sinensis* (MFLUCC 17-1344, ex-type) showed that the LSU and SSU gene has no nucleotide differences, while the TEF gene of *M.sinensis* (MFLUCC 17-1344, ex-type) is unavailable in NCBI (Hyde et al. 2018). The comparison of the TEF nucleotides between the new collection and another strain of *M.sinensis* (MFLUCC 17-2606) resulted in 0.3% differences (3/873 bp, without gaps) (Senwanna et al. 2021). This study first discovered *M.sinensis* on *Aquilaria* sp. in China. Therefore, we introduce our new collection as a new host and geographical record of *M.sinensis* based on both morphological study and phylogenetic analyses.

#### ﻿ Melomastia yunnanensis

Taxon classificationFungiDyfrolomycetalesPleurotremataceae

T.Y. Du, K.D. Hyde, Tibpromma & Karun. sp. nov.

7F0CEEFC-4872-575B-9E54-43F3A9DD51BA

856408

Facesoffungi Number: FoF16959

Fig. 4

##### Etymology.

Named after the type location “Yunnan, China”.

##### Holotype.

GMB-W 1007

##### Description.

***Saprobic*** on a dead branch of *Aquilaria* sp. ***Sexual morph*: *Ascomata*** (excluding neck) 400–500 µm high × 300–480 µm diam. (x– = 458 × 395 µm, n = 10), solitary, scattered to gregarious, immersed to erumpent through host tissue, globose, black, carbonaceous, ostiolate. ***Ostiolar canal*** 100–160 µm high × 120–230 µm wide (x– = 130 × 184 µm, n = 10), central, black, conical, carbonaceous, filled with hyaline sparse periphyses. ***Peridium*** 25–75 µm wide (x– = 55 µm, n = 10), comprising of dense, several layers of brown to dark brown, thick-walled cells of ***textura angularis*** to ***textura prismatica***. ***Hamathecium*** comprising 2.5–7.5 µm wide, numerous filamentous, filiform, septate, sometimes branched, hyaline pseudoparaphyses, attached to the base and between the asci, embedded in a gelatinous matrix. ***Asci*** 180–220 × 7.5–10.5 µm (x– = 195.5 × 9 µm, n = 30), bitunicate, 8-spored, cylindrical, short pedicel, thickened and rounded apex, with an obvious ocular chamber. ***Ascospores*** 20–24.5 × 6–8 µm (x– = 22.5 × 7 µm, n = 30), overlapping-uniseriate, hyaline, when ascospores gather together, they appear light yellow, mostly 6–8-septate at maturity, mostly 7-septate, cylindrical, with rounded ends, slightly constricted at the septum, often similar width of cells with several small guttules, not surrounded by a mucilaginous sheath. ***Asexual morph***: Undetermined.

**Figure 4.**
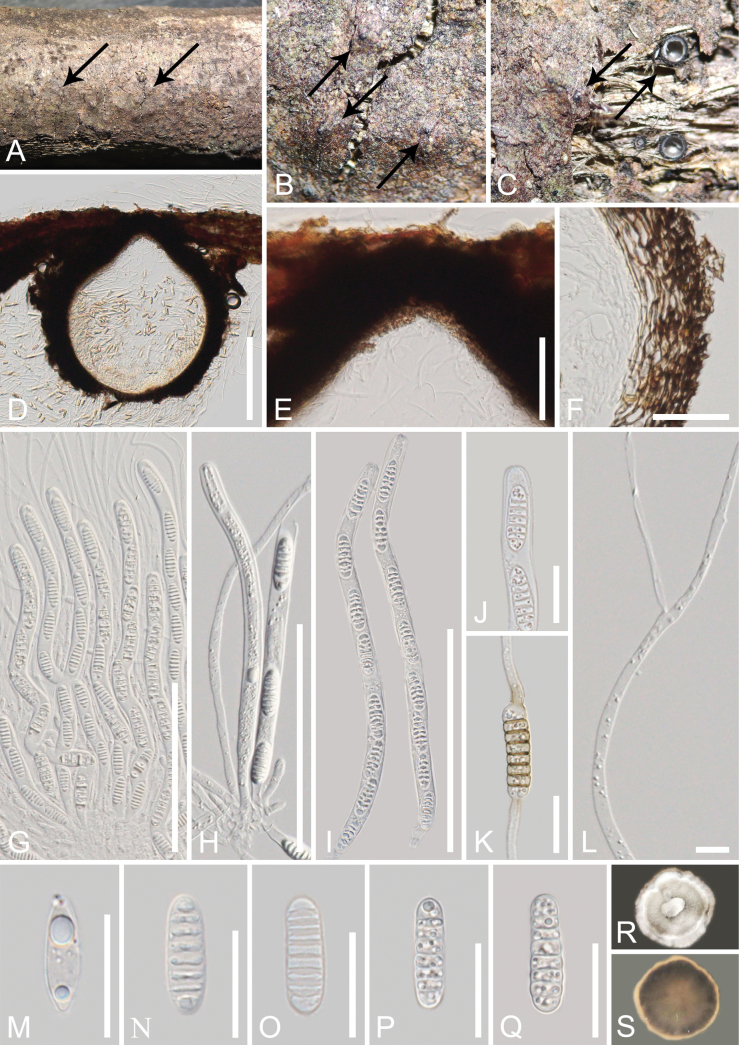
*Melomastiayunnanensis* (GMB-W 1007, holotype) **A–C** appearance of ascomata on the host (the arrows indicate ascomata) **D** vertical sections through the ascoma **E** ostiole **F** peridium **G–I** asci **J** asci ocular chamber **K** germinated ascospore **L** pseudoparaphyses **M–Q** ascospores **R, S** colonies on PDA obverse and reverse view. Scale bars: 200 µm (**D**); 100 µm (**G–I**); 50 µm (**E, F**); 20 µm (**J, K, M–Q**); 10 µm (**L**).

##### Culture characteristics.

Ascospores germinated on PDA after 24 hours, germ tubes were produced from both ends, germinated ascospores appear light brown. ***Colonies*** on PDA reaching 2 cm diam., after two weeks at 23–28 °C. Colonies obverse: dense, circular, umbonate, gray at the center, cream, and entire edge. Colonies reverse: gray brown, light brown at the margin.

##### Material examined.

China • Yunnan Province, Xishuangbanna, Jinghong City, Naban River Nature Reserve, 22°7'51"N, 100°40'21"E, on a dead branch of *Aquilaria* sp. (Thymelaeaceae), 14 September 2021, Tianye Du, YNA51 (GMB-W 1007, holotype), ex-type, GMBCC1009, other living culture, GZCC 23-0621.

##### Notes.

In the phylogenetic analyses, our new collection, *M.yunnanensis* formed a sister branch with *M.sinensis* (MFLUCC 17-1344, MFLUCC 17-2606, MFLU 17-0777, and GMBCC1008) in *Melomastiasensu lato* with a 100% ML/1.00 PP bootstrap support (Fig. 1). NCBI BLASTn searches of our collection *M.yunnanensis* showed 99.23% similarity to *M.sinensis* (MFLUCC 17-2606) in the LSU sequence, 98.92% similarity to *M.thamplaensis* (AND9) in the SSU sequence, and 96.34% similarity to *M.sinensis* (MFLUCC 17-2606) in the TEF sequence. Our new collection, *M.yunnanensis* shares similar morphology with *M.sinensis* in cylindrical and septate ascospores. However, *M.sinensis* differs from *M.yunnanensis* in having superficial, semi-immersed to immersed ascomata, cylindrical or conical ostiolar canal, and unbranched pseudoparaphyses (Hyde et al. 2018), while our *M.yunnanensis* has immersed ascomata, conical ostiolar canal, and pseudoparaphyses sometimes branched. In addition, the nucleotide base pair differences between our new collection *M.yunnanensis* (GMBCC1009, ex-type) and *M.sinensis* (MFLUCC 17-1344, ex-type) showed the LSU gene has 0.5% nucleotide differences (4/760 bp, without gaps), the SSU gene has 0.5% nucleotide differences (4/813 bp, without gaps), while the TEF gene of *M.sinensis* (MFLUCC 17-1344, ex-type) is unavailable (Hyde et al. 2018). We compared the TEF nucleotides between the new collection and another collection of *M.sinensis* (MFLUCC 17-2606), which resulted in 3.8% differences (33/873 bp, without gaps) (Senwanna et al. 2021). Therefore, we introduce our new collection, *M.yunnanensis*, as a new species on a dead branch of *Aquilaria* sp. from terrestrial habitats in China, based on both morphological study and phylogenetic analyses following the guidelines of Maharachchikumbura et al. (2021).

## ﻿Discussion

Based on the morphological study and phylogenetic analyses, this study identifies, describes, and introduces two new species, *Melomastiaguangdongensis* and *M.yunnanensis*, and a new host and geographical record of *M.sinensis* from *Aquilaria* spp. These findings significantly contribute to the understanding of the diversity and distribution of agarwood resin-producing tree-associated fungi.

Our phylogenetic analysis based on LSU, SSU, and TEF also showed that the results are similar to those of Kularathnage et al. (2023) and Xu et al. (2024), who have divided *Melomastia* into two clades, *Melomastiasensu lato* and *Melomastiasensu stricto.* However, the majority of species are clustered in *Melomastiasensu lato*, and only 20 out of 66 listed records in Index Fungorum (2024) have available sequences, posing a challenge for the study of phylogenetic analysis in this genus. To address this, we believe it is necessary to explore and collect more samples of new and known species of *Melomastia* and supplement our research with molecular studies. In addition, relevant information about *Melomastia*, such as life mode, habitat, host, geographical location, and ecological niche, must be collected and analyzed to enhance our knowledge of this genus.

Morphologically, most species in *Melomastia* have fusiform or ellipsoidal ascospores, while two species (*M.marinospora* and *M.sinensis*) show cylindrical ascospores (Li et al. 2022). Previously, the ascospores of this genus are usually reported 3-septate (e.g. *M.aquatica*, *M.clematidis*, *M.distoseptata*, *M.fusispora*, *M.maolanensis*, *M.marinospora*, *M.oleae*, *M.sichuanensis*, *M.thamplaensis*, and *M.winteri*) (Li et al. 2022). Current studies as more new taxa were introduced into this genus reveal multi-septate ascospores, while these taxa with similar characteristics do not cluster together on the phylogenetic tree (Fig. 1), such as *M.mangrovei* (7–9-septate, no molecular data available in NCBI), *M.phetchaburiensis* (1–10-septate, in *Melomastiasensu lato*), *M.rhizophorae* (4–6-septate, in *Melomastiasensu stricto*), *M.sinensis* (6–7-septate, in *Melomastiasensu lato*), and *M.thailandica* (3–5-septate, in *Melomastiasensu stricto*) (Li et al. 2022). In this study, *M.guangdongensis* shows the fusiform with 3-septate ascospores, while *M.yunnanensis* shows the cylindrical with 6–8-septate ascospores, both of these new taxa belong to *Melomastiasensu lato.* Therefore, more studies are needed to discuss the morphological and phylogenetic connections of this genus. In addition, in this study, we also found *Melomastia* from the same host genus *Aquilaria*, but when we compare ascomata, semi-immersed to immersed ascomata in *M.guangdongensis* and *M.sinensis*. In contrast, ascomata of *M.yunnanensis* are immersed to erumpent through host tissue. Further research is needed to explore whether the attachment mode of ascomata on the substrate is influenced by the host, environment, or other factors.

In recent years, many studies on saprobic fungi in economic crops, such as rice, sugarcane, rubber, coffee, mango, and macadamia nuts, have been published (Yang et al. 2022, Lu et al. 2024, Tian et al. 2024, Xu et al. 2024, Zhang et al. 2024). However, there is a noticeable lack of research on saprobic fungi in *Aquilaria* spp. This study introduces three saprobic fungal taxa, expanding the previous record of 28 saprobic fungi associated with *Aquilaria* to 31. It also highlights the urgent need for further, more in-depth investigations. We believe that future studies with a broader geographical range will be crucial in enhancing our understanding of the distribution and diversity of fungi in *Aquilaria*.

## Supplementary Material

XML Treatment for Melomastia guangdongensis

XML Treatment for Melomastia sinensis

XML Treatment for Melomastia yunnanensis

## ﻿Additional information

### Author ORCIDs

Tian-Ye Du https://orcid.org/0000-0003-2105-1803

Samantha C. Karunarathna https://orcid.org/0000-0001-7080-0781

Saowaluck Tibpromma https://orcid.org/0000-0002-4706-6547

Kevin D. Hyde https://orcid.org/0000-0002-2191-0762

Somrudee Nilthong https://orcid.org/0000-0002-7454-5826

Ausana Mapook https://orcid.org/0000-0001-7929-2429

Xiang-Fu Liu https://orcid.org/0000-0003-0100-2094

Dong-Qin Dai https://orcid.org/0000-0001-8935-8807

Chen Niu https://orcid.org/0009-0008-4633-9719

Abdallah M. Elgorban https://orcid.org/0000-0003-3664-7853

Ekachai Chukeatirote https://orcid.org/0000-0002-9968-5841

Hao-Han Wang https://orcid.org/0000-0002-2128-7894

### Data availability

All of the data that support the findings of this study are available in the main text.
